# Identification of Tumor Antigens in Ovarian Cancers Using Local and Circulating Tumor-Specific Antibodies

**DOI:** 10.3390/ijms222011220

**Published:** 2021-10-18

**Authors:** Jessica Da Gama Duarte, Luke T. Quigley, Anna Rachel Young, Masaru Hayashi, Mariko Miyazawa, Alex Lopata, Nunzio Mancuso, Mikio Mikami, Andreas Behren, Els Meeusen

**Affiliations:** 1 Olivia Newton-John Cancer Research Institute, School of Cancer Medicine, La Trobe University, Heidelberg, VIC 3084, Australia; jessica.duarte@onjcri.org.au (J.D.G.D.); luke.quigley@onjcri.org.au (L.T.Q.); andreas.behren@onjcri.org.au (A.B.); 2 La Trobe Institute for Molecular Science, La Trobe University, Bundoora, VIC 3086, Australia; aryoung@unimelb.edu.au; 3 School of Medicine, Tokai University, Isehara-City 259-1193, Japan; hayashimasaru0620@hotmail.com (M.H.); marikom@tokai-u.jp (M.M.); mmikami@is.icc.u-tokai.ac.jp (M.M.); 4 CancerProbe Pty Ltd., Prahran, VIC 3181, Australia; alex.lopata15@gmail.com (A.L.); nunziom@gmail.com (N.M.); 5 Department of Medicine, University of Melbourne, Parkville, VIC 3010, Australia; 6 School of Science, Psychology & Sport, Federation University, Mount Helen, VIC 3841, Australia

**Keywords:** ovarian cancer, biomarkers, antibody-secreting B cells, circulating antibodies, protein microarrays, diagnosis

## Abstract

Ovarian cancers include several disease subtypes and patients often present with advanced metastatic disease and a poor prognosis. New biomarkers for early diagnosis and targeted therapy are, therefore, urgently required. This study uses antibodies produced locally in tumor-draining lymph nodes (ASC probes) of individual ovarian cancer patients to screen two separate protein microarray platforms and identify cognate tumor antigens. The resulting antigen profiles were unique for each individual cancer patient and were used to generate a 50-antigen custom microarray. Serum from a separate cohort of ovarian cancer patients encompassing four disease subtypes was screened on the custom array and we identified 28.8% of all ovarian cancers, with a higher sensitivity for mucinous (50.0%) and serous (40.0%) subtypes. Combining local and circulating antibodies with high-density protein microarrays can identify novel, patient-specific tumor-associated antigens that may have diagnostic, prognostic or therapeutic uses in ovarian cancer.

## 1. Introduction

Ovarian tumors are the most lethal of all female reproductive cancers, with over 70% mortality within five years of diagnosis [1]. Tumor progression is characteristically accompanied by few or ambiguous symptoms, resulting in patients often presenting with advanced metastatic disease and a poor prognosis. By contrast, patients diagnosed with early disease exhibit survival rates above 90%, with disease often cured by surgery alone [2]. For this reason, early detection is widely believed to be the best strategy to improve patient outcomes, but no screening tests are currently available [2,3].

Ovarian cancer is now recognized to be a collective term for several disease subtypes (including serous, mucinous, clear cell and endometrioid carcinomas) with different tissue origins, hereditary susceptibilities and gene expression profiles [4,5,6]. New biomarkers and more targeted treatment options that take into account the high degree of heterogeneity associated with ovarian cancer are therefore required [7,8].

Tumor antigens are expressed by malignant cells and can arise through several mechanisms, including mutations, post-translational modifications and aberrant tissue expression [9,10]. Irrespective of their initiation, tumor antigens can induce highly specific antibodies that, in some cases, can be detected several years prior to the manifestation of clinical symptoms [11,12,13], making them attractive targets for early diagnosis. Antibodies enable the identification of patient-specific tumor antigens [9,10], which are targets for immunotherapies such as CAR-T cell therapy and vaccines [14]. Antibody profiles have also been shown to predict immune-related adverse events during treatment with immune checkpoint inhibitors in melanoma [15,16].

Antibody generation requires the induction of antibody-secreting B cells (ASCs) in tissue-draining lymph nodes, with the subsequent release of antibodies into the blood. Previous studies have shown that ASCs recovered from tissue-draining lymph nodes are highly enriched for disease-related antigens compared to those in the blood [17,18] and can be used to identify novel tumor antigens [19,20]. To identify the cognate antigens recognized by tumor antigen-induced antibodies, high-density protein microarrays have emerged as useful tools, although challenges with cross-reactivity and high background are common [21].

In this study, we hypothesized that tumor antigen-enriched antibodies harvested from ASCs present in the lymph nodes (ASC probes) of ovarian cancer patients can be used to identify patient-specific tumor antigens. We screened these ASC probes using two commercial high-density protein microarrays and identified 50 candidate antigens. A custom 50-antigen array was constructed and used to screen sera from a separate patient cohort to investigate the diagnostic potential.

## 2. Results

### 2.1. Antibody Reactivity Profiles of ASC probes against Ovarian Cancer Cell Line

**Figure 1:**
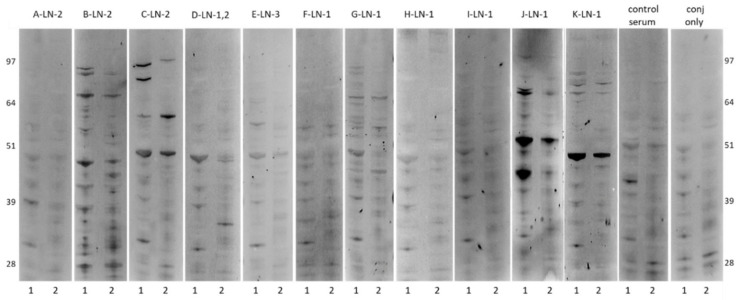
Western blots of soluble (1) and insoluble (2) fractions of OVCAR3 cell extracts, screened with selected ASC probes from 11 ovarian cancer patients (A–K). Molecular weight markers on the far left and right are recorded from pre-stained markers. Control lanes on the right are screened with serum from healthy women (control serum) or secondary antibody conjugate only (conj only).

ASC probes were generated from one to three separate pelvic lymph nodes (LN1, LN2, LN3) of 11 ovarian cancer patients (A–K). The IgG concentrations of ASC probes ranged from 0.04 µg/mL to 1.05 µg/mL, excluding an outlier (C-LN1, 15.00 µg/mL) (Table 1). Western blots of ASC probes with the highest IgG content were performed against soluble (1) or insoluble (2) extracts of an ovarian cancer cell line (OVCAR3), and showed unique and shared patterns of band reactivity with higher reactivity seen in soluble fractions (Figure 1), as well as instances of no reactivity (A-LN2, H-LN1).

### 2.2. Candidate Antigen Selection Using High-Density Protein Microarrays

This study utilized two commercial high-density protein microarrays—the CDI HuProt^TM^ array (16,086 human proteins and protein isoforms) and the Sengenics CTA protein array (257 cancer-testis and -associated antigens)—to screen ASC probes and matched sera from cohort 1 (*n* = 11) to identify candidate antigens.

Using the CDI HuProt^TM^ array, pooled healthy (*n* = 2) and benign (*n* = 2, adenomas) serum controls showed detectable autoantibodies (Z-score > 3) against 122 and 177 antigens (133 unique to benign disease), respectively, which were used to eliminate non-specific hits and determine cancer-specificity of candidate antigens detected by ASC probes. A total of 45 cancer-specific antigens showed high ASC probe reactivity (RFU > 6000) and no reaction in healthy and benign sera (Table 2). Overlapping cancer-specific antigen specificities were seen for a subset of antigens in all cases with matched ASC probes and sera.

Using the Sengenics CTA protein array, pooled benign controls (*n* = 2, adenomas) showed detectable autoantibodies (Z-score > 3) against five antigens (AURKA, CTAG1A, CYP450 3A4, MAGEA4 and RAF1). A total of 25 cancer-specific antigens showed high ASC-probe reactivity (Z-score > 3) and no reaction in benign serum (Table 3). Overlapping cancer-specific antigen specificities were seen for a subset of antigens in two out of five cases with matched ASC probes and sera.

### 2.3. Diagnostic Screen Using a Custom Protein Microarray

Upon candidate antigen identification using the above described commercially available high-density arrays, a 50-antigen custom protein array was generated via CDI (Table S1). This custom array consisted of 41 cancer-specific antigens selected from the highest-reacting antigens for each ASC probe and nine benign disease-specific antigens (AURKA, CROCCP2, CTAG1A, MAGEA4, MAGEA9, PALM2, PMEPA1, SMYD5_frag and USP14) selected from the highest-reacting antigens in benign serum (and no reaction in healthy serum) on the high-density protein microarrays. This custom protein array was used to screen serum from cohort 2 (*n* = 80) to determine its diagnostic potential.

**Figure 2:**
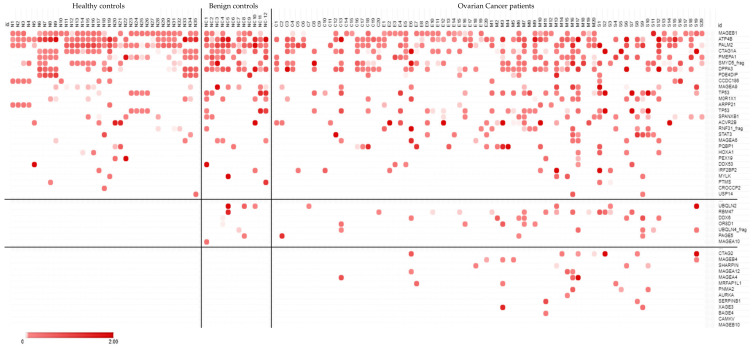
Antigen reactivities identified with ovarian cancer cohort 2 using the custom protein array. Healthy controls (*n* = 35), benign controls (*n* = 12) and ovarian cancer patients (*n* = 80) were screened using the CDI custom protein array. Data not shown for PLEKHA8 and PRM2 due to abundant uniform reactivity observed across all healthy, all benign and almost all cancer samples (*n* = 78/80, *n* = 79/80 respectively). Data are shown as Z-scores, with scale bar set from 0 (white) to 2 (bright red), and all values with a Z-score < 0 are in white. Ovarian cancer subtypes are classified as clear cell (C1-20), endometrioid (E1-20), mucinous (M1-20) and serous (S1-20).

Antibody-profiling serum from healthy controls (*n* = 35), benign controls (*n* = 12) and ovarian cancer patients across four disease subtypes (*n* = 80) using the custom protein array showed detectable autoantibodies (Z-score > 0) against all antigens except CAMKV and MAGEB10 (*n* = 48/50). Two antigens (*n* = 2/50, PLEKHA8 and PRM2) showed abundant uniform reactivity across all healthy, all benign and virtually all cancer samples (*n* = 78/80, *n* = 79/80 respectively) and were excluded from subsequent analyses. Antigen reactivities were either shared across all healthy, benign and cancer groups (*n* = 28/48), shared between benign and cancer groups (*n* = 7/48) or exclusive to cancer patients (*n* = 11/48) (Figure 2). The latter included six cancer-testis antigens (CTAG2, MAGEB4, MAGEA12, MAGEA4, XAGE3, BAGE4), and five other tumor-associated antigens (SHARPIN, MRFAP1L1, PNMA2, AURKA, SERPINB1). Antigen reactivity against these tumor-associated antigens identified 28.8% of all ovarian cancer patients (*n* = 23/80), particularly those with mucinous (*n* = 10/20) and serous (*n* = 8/20) disease subtypes. Correlation to clinical features was not performed due to the limited patient numbers within disease subtypes.

### 2.4. Candidate Gene Expression Using the Cancer Genome Atlas (TCGA)

**Figure 3:**
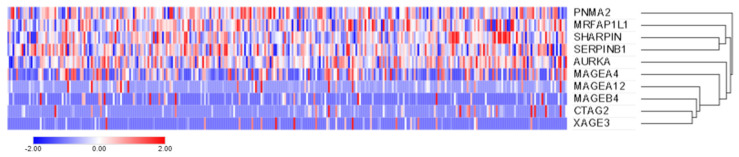
Gene expression of candidate tumor-associated and cancer-testis antigens in a separate ovarian cancer TCGA dataset. Heat map representing the hierarchical clustering using Euclidean distance of 307 ovarian serous cystadenocarcinomas (TCGA dataset) based on *CTAG2, MAGEB4, SHARPIN, MAGEA12, MAGEA4, MRFAP1L1, PNMA2, AURKA, SERPINB1* and *XAGE3* mRNA expression. mRNA expression is shown as Z-scores (RNA Seq V2 RSEM) with scale bar set from −2 (blue) to 2 (red).

To determine if the prevalence of antibodies against the identified tumor-associated antigens (CTAG2, MAGEB4, SHARPIN, MAGEA12, MAGEA4, MRFAP1L1, PNMA2, AURKA, SERPINB1 and XAGE3) is reflected in the prevalence of the respective gene expression across ovarian cancer patients, we assessed a separate serous ovarian cancer cohort accessible via TCGA (*n* = 307/606 with available mRNA expression data) for gene expression (no gene expression data were available for *BAGE4*). While the tumor-associated antigens (*SHARPIN, MRFAP1L1, PNMA2, AURKA, SERPINB1*) were expressed at the transcript level in all ovarian cancer samples (*n* = 307/307, 100%), expression was less prevalent for the cancer-testis antigens (*MAGEA4*: *n* = 224/307, 73%; *MAGEA12*: *n* = 102/307, 33.2%; *MAGEB4*: *n* = 78/307, 25.4%; *CTAG2*: *n* = 76/307, 24.8%; *XAGE3*: *n* = 48/307, 15.6%, Figure 3).

In the serous ovarian cancer patients of cohort 2 (*n* = 20/80), detectable antibodies (Z-scores > 0) were seen against CTAG2 (*n* = 3/20, 15%), SHARPIN (*n* = 2/20, 10%), PNMA2 (*n* = 2/20, 10%), MAGEB4 (*n* = 1/20, 5%), MRFAP1L1 (*n* = 1/20, 5%), SERPINB1 (*n* = 1/20, 5%) and XAGE3 (*n* = 1/20, 5%). When compared to the above-reported gene expression, antibody prevalence was lower than expected for all antigens.

## 3. Discussion

Identifying novel diagnostic biomarkers or therapeutic targets in ovarian cancer, particularly those that prevail over the heterogeneity seen across disease subtypes, has proven challenging to date and presents an area of unmet clinical need. Antibodies against tumor antigens have been suggested as promising biomarkers for ovarian cancer [22]. In this study, we aimed to use ASC probes (antibodies harvested from ASCs recovered from tissue-draining lymph nodes) as a source of antibodies enriched against tumor antigens. Here, we showed that ASC probes can be screened on high-density protein microarrays to identify candidate cognate tumor antigens, yielding a proportion of overlapping antigen specificities when compared to matched serum. Moreover, this enabled the identification of novel tumor-associated antigens that may be used to detect ovarian cancers or targeted therapeutically.

In this study, most ASC probes isolated from a small cohort of ovarian cancer patients (*n* = 9/11) reacted to extracts of a high-grade serous ovarian cancer cell line (OVCAR3) with varying intensity. While antigen reactivity was lower than previously detected in breast cancer, both shared and unique patterns were also seen [19]. Interestingly, higher antigen reactivities were seen in soluble cell fractions containing cytoplasmic antigens, when compared to insoluble fractions mostly including membranous antigens. This is in agreement with previous studies, and has been associated with immunosuppression [23,24]. Given that the utility and origin of ovarian cancer cell lines have previously been questioned [25], reactivity against cell line extracts from additional ovarian cancer subtypes was not investigated in this study.

Screening the ASC probes on two high-density protein microarrays, the CDI HuProt^TM^ array and the Sengenics CTA protein array, led to the identification of 41 candidate cancer-specific antigens. Whilst shared antigen reactivities were seen across patients, each patient exhibited a unique antibody profile, as was previously observed in breast cancer [19]. Matched sera for a subset of patients revealed instances of shared antigen reactivities in all patients between the ASC probe and serum pairs, as per previous studies [19,26]. The CDI HuProt^TM^ array showed broader antigen reactivity in serum when compared to matched ASC probes, most likely due to the more targeted, temporal lymph node response in the latter. A subset of antigens was present on both protein microarrays, and detectable antibodies against one antigen (TP53) were simultaneously seen in ASC probes from patient J (J-LN1).

A custom array was constructed using the above identified 41 cancer-specific and 9 benign candidate antigens and used to screen a separate ovarian cancer cohort (*n* = 80), along with age and gender-matched healthy (*n* = 35) and benign (*n* = 12) controls. This diagnostic screen identified 28.8% of patients across disease subtypes and led to the identification of 11 tumor-associated antigens (CTAG2, MAGEB4, SHARPIN, MAGEA12, MAGEA4, MRFAP1L1, PNMA2, AURKA, SERPINB1, XAGE3 and BAGE4) with detectable antibodies exclusive to cancer patients, including several cancer-testis antigens. These data are comparable to previous antibody profiling studies in serous ovarian cancers, which reported sensitivities ranging from of 23 to 45% using multi-antigen signatures [27,28]. As a means of validating our findings, corresponding gene expression was investigated in a separate cohort (*n* = 307) accessible via TCGA, confirming expression at the transcript level ranging from 15.6% to 100% of serous ovarian cancer samples. While antibody profiling of these candidate antigens was not performed in other cancers, it is unlikely that these antigens are ovarian cancer-specific given the abundant expression reported across cancer tissues (TCGA).

Cancer-testis antigen expression has previously been reported in ovarian cancer, with specific associations to tumor progression, prognosis and therapeutic resistance, thereby supporting their potential uses as novel diagnostic biomarkers or immunotherapeutic targets [29]. Previous studies that investigated MAGEA4 in ovarian cancer reported tissue expression in 57% of serous carcinomas [30] and 47% of epithelial ovarian cancer patients [31], serum levels in 22% of primary ovarian cancer patients [32] and autologous antibodies in 9% of epithelial ovarian cancer patients [31]. Of note, all studies reported correlation between MAGEA4 and poor patient survival. Similarly, CTAG2 (alias LAGE-1, sharing 94% homology with NY-ESO-1 [32]) was expressed in 21% of epithelial ovarian cancer tissues, 30% of which also had detectable autologous antibodies [33].

Combining the use of ASC probes with sensitive, high-density protein microarrays is an attractive method to identify novel, patient-specific tumor-associated antigens that may have diagnostic, prognostic or therapeutic uses in ovarian cancer. Clinical evaluation is warranted to investigate the further applicability of these findings.

## 4. Materials and Methods

### 4.1. Patient Cohorts

Specimens from two separate cohorts of ovarian cancer patients were used in this study. Cohort 1 (*n* = 11) included multiple surgically resected pelvic lymph nodes and matched serum samples, while cohort 2 (*n* = 80) included serum samples from clear cell, endometrioid, mucinous and serous carcinoma subtypes (Table 4). In addition, serum from healthy females (*n* = 35) and those with benign gynecological conditions (*n* = 12) was also obtained (Table 5). This study was conducted with ethical approval from the La Trobe University Human Ethics Committee (HEC17106) and the Research Ethics Committee of Tokai University Hospital (IRB registration number: 09R-082, 15R-253), including the use of clinical information and patient samples.

### 4.2. Generation of ASC Probes and IgG Quantification

Single cell suspensions were prepared from separate lymph node tissues, cultured for five days, and the supernatants collected (ASC probes) and used as previously described [18,19]. The total IgG content of ASC probes was measured using a human IgG quantification kit (FastElisa; R&D Biotech, Besancon, France).

### 4.3. Western Blotting

Western Blotting of ASC probes against soluble and insoluble extracts of a high-grade serous ovarian cancer cell line (OVCAR3) was performed as previously described [19].

### 4.4. Antibody Profiling Using Protein Microarrays

**Figure 4:**
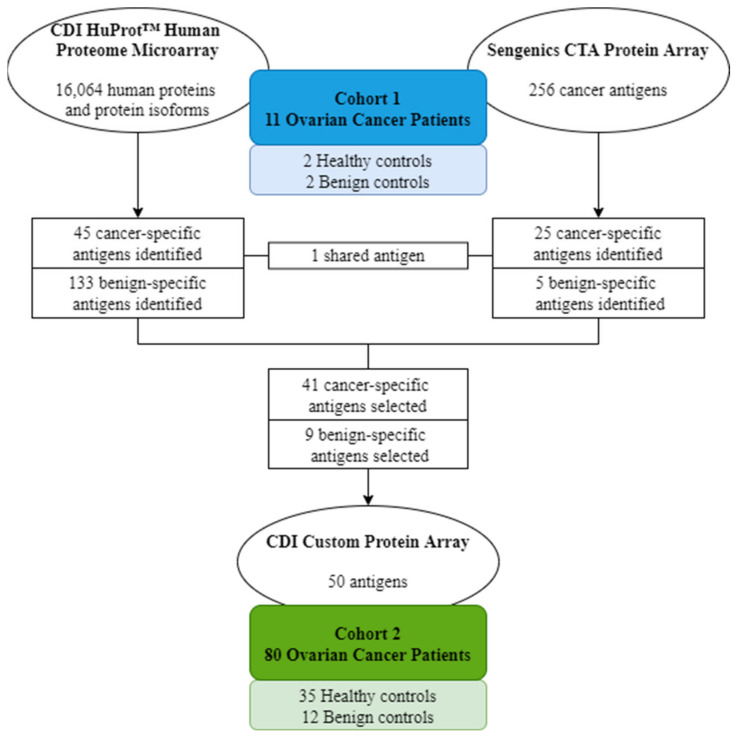
Study design diagram. Ovarian cancer cohort 1 and selected healthy and benign controls were screened using the CDI HuProt^TM^ array and the Sengenics CTA protein array, which led to the identification of 50 candidate antigens. A custom array was developed and used to screen ovarian cancer cohort 2, as well as healthy and benign controls.

ASC probes and matched sera from ovarian cancer cohort 1 (*n* = 11), as well as serum from healthy (*n* = 2) and benign (*n* = 2) controls, were screened using the CDI Human Proteome (HuProt^TM^) array (CDI Laboratories Inc., Mayaguez, PR, USA) and the Sengenics Cancer-Testis Antigens (CTA) protein array (Sengenics Corporation, Singapore), as per the manufacturers’ instructions. Both arrays were used to ensure the maximum coverage of the human proteome and tumor antigens relevant to cancer. The CDI HuProt^TM^ array included 16,064 human proteins and protein isoforms and was used to screen ASC probes at a 1:2 dilution and serum at a 1:500 dilution. The Sengenics CTA protein array included 256 cancer-testis and associated antigens and was used to screen ASC probes at a 1:2 dilution and serum at a 1:800 dilution. While some protein content was shared between the HuProt^TM^ and CTA arrays (189 antigens), the protein cloning and expression methods used by each array platform differed (N-terminal GST-His-tagged proteins produced using the pEGH-A expression vector in a *Saccharomyces cerevisiae* strain versus BCCP-tagged fusion proteins produced in SF9 insect cells and biotinylated in vivo, respectively) [21]. These data were used to identify candidate antigens relevant to ovarian cancer, and a custom protein array including 50 candidate antigens (Table S1) was generated via CDI (CDI Laboratories Inc.). Serum samples from ovarian cancer cohort 2 (*n* = 80), as well as all healthy (*n* = 35) and benign (*n* = 12) controls, were screened at a 1:500 dilution using the CDI custom protein array, as per the manufacturer’s instructions (Figure 4).

### 4.5. Gene Expression Profiling Using the Cancer Genome Atlas (TCGA)

Gene expression profiling using the identified tumor-associated antigens was investigated using publicly available ovarian cancer datasets accessible via TCGA research network (http://cancergenome.nih.gov/ (accessed on 3 August 2021)) and analyzed using the cBioPortal [34,35]. The TCGA dataset used was that of ovarian serous cystadenocarcinoma (TCGA, Firehose legacy), consisting of 606 samples (594 patients). Absolute mRNA transcript values were used to determine the levels of expression across TCGA patient samples, with any transcript level above zero considered “positive” for expression. The mRNA expression levels of the respective genes are shown as Z-scores (RNA Seq V2 RSEM) and represented in a log2 scale.

## Figures and Tables

**Table 1:** IgG concentration (µg/mL) of ASC probes isolated from 11 ovarian cancer patients (columns A–K), separated by individual lymph node sources.

Patient LN/µg/mL	A	B	C	D	E	F	G	H	I	J	K
**LN1**	0.14	0.12	15.00	0.28	0.24	0.18	0.45	0.26	0.47	1.05	0.39
**LN2**	0.28	0.34	0.58		0.65	0.04		0.04		0.06	
**LN3**			0.88		0.33	0.14					

**Table 2:** Antigen reactivities identified with ovarian cancer cohort 1 using CDI HuProt^TM^ arrays. Ovarian cancer ASC probes (*n* = 11, A–K) and selected matched sera (*n* = 5) were screened using CDI HuProt^TM^ arrays.

ASC Probes	No. of Cancer-Specific Hits (RFU > 6000)	Top Hits (RFU > 6000)	Serum	No. of Positive Hits (Z-Score > 3)	No. of Cancer-Specific Hits (Z-Score > 3)	Shared ASC Probe and Serum Cancer-Specific Hits
A—LN2	3	PLEKHA8 PEX19 PNMA2	-	-	-	-
B—LN2	0	-	B	100	52	-
C—LN3	9	M0R1X1 IRF2BP2 USP5 PDE4DIP GTF2I EVC MFSD5 SLC25A22 GAD1	C	134	92	IRF2BP2 USP5 PDE4DIP GTF2I GAD1
D—LN1,2	5	ATP4B PTMS GAGE10 SNX33 SLC25A22	-	-	-	-
E—LN2	4	OR8D1 ZIC2 PAGE5 CAMKV	-	-	-	-
F—LN1	3	PLEKHA8 MAGEA4 SLC25A22	-	-	-	-
G—LN1	8	MAGEA4 STAT3 MAGEB10 MAGEA10 DDX6 MAGEA12 SERPINB1 MAGEA6	G	95	65	MAGEA4 STAT3 MAGEB10 MAGEA10 DDX6 MAGEA12
H—LN1	1	MYLK	-	-	-	-
I—LN1	0	-	-	-	-	-
J—LN1	13	TP53 UBQLN4_frag UBQLN2 SHARPIN RBM47 RNF31_frag HOXA1 CCDC97 ZFYVE19 UBQLN1 RBCK1 DDX53 KCTD18	J	105	84	TP53 UBQLN4_frag UBQLN2 SHARPIN RBM47 RNF31_frag HOXA1 ZFYVE19 UBQLN1 RBCK1 DDX53 KCTD18
K—LN1	4	PQBP1 MRFAP1L1 ARPP21 DDX53	K	136	86	PQBP1 MRFAP1L1 ARPP21 DDX53

**Table 3:** Antigen reactivities identified with ovarian cancer cohort 1 using Sengenics CTA protein arrays. Ovarian cancer ASC probes (*n* = 11, A–K) and selected matched sera (*n* = 5) were screened using Sengenics CTA protein arrays.

ASC Probes	No. of Cancer-Specific Hits (Z-Score > 3)	Top Hits (Z-Score > 3)	Serum	No. of Cancer-Specific Hits (Z-Score > 3)	Top Hits (Z-Score > 3)	Shared ASC Probe and Serum Cancer-Specific Hits
A—LN2	2	BAGE4 DSCR8/MMA1	-	-	-	-
B—LN2	2	BAGE4 DSCR8/MMA1	B	1	SPANXN4	0
C—LN1	4	BAGE4 CTNNB1 DMRTC2 DSCR8/MMA1	C	1	ACVR2A	0
C—LN2	6	MAGEA4v3 MAGEB1 MAGEB2 OIP5 PRM2 XAGE3aV1				
C—LN3	1	DSCR8/MMA1				
D—LN1,2	2	BAGE3 p53L344P	-	-	-	-
E—LN2	2	DSCR8/MMA1 MAGEA4v3	-	-	-	-
E—LN3	4	DMRTC2 DSCR8/MMA1 GRWD1 MAGEB4				
F—LN1	4	BAGE3 CCNA1 DSCR8/MMA1 MAGEA4v3	-	-	-	-
G—LN1	1	MAGEA4v2	G	2	MAGEA10 MAGEA4v2	MAGEA4v2
H—LN1	2	BAGE4 DSCR8/MMA1	-	-	-	-
I—LN1	3	BAGE4 DSCR8/MMA1 MAGEA4v3	-	-	-	-
J—LN1	9	p53C141Y p53K382R p53M133T p53S15A p53S392A p53S46A p53S6A p53T18A TP53	J	8	p53K382R p53M133T p53S15A p53S392A p53S46A p53S6A p53T18A TP53	p53K382R p53M133T p53S15A p53S392A p53S46A p53S6A p53T18A TP53
K—LN1	1	BAGE4	K	1	CTAG2	0

**Table 4:** Summary of ovarian cancer patient characteristics across both cohorts. This includes sample number, age, body mass index (BMI), ethnicity, risk factors, disease stage, subtype and cancer antigen-125 (CA-125) levels.

	Cohort 1 (*n* = 11)	Cohort 2 (*n* = 80)
Sample types	Pelvic lymph nodes, serum	Serum
Age—years		
Median	52	54
Range	31–69	23–83
BMI—no.		
Median	-	24
Range	-	18–41
Ethnicity—no. (%)		
Asian	11 (100.0)	24 (30.0)
Caucasian	-	56 (70.0)
Risk Factors—no. (%)		
Smoking	-	6 (7.5)
Familial cancers	-	21 (26.3)
Hypertension	-	22 (27.5)
Diabetes mellitus	-	3 (3.8)
Stage—no. (%)		
I	3 (27.3)	49 (61.2)
II	1 (9.1)	11 (13.8)
III	7 (63.6)	18 (22.5)
IV	0 (0.0)	2 (2.5)
Ovarian Cancer Subtype—no. (%)		
Clear cell	2 (18.2)	20 (25.0)
Endometrioid	0 (0.0)	20 (25.0)
Mucinous	2 (18.2)	20 (25.0)
Serous	5 (45.4)	20 (25.0)
Mixed	2 (18.2)	0 (0.0)
CA-125 levels—U/mL		
Median	441	317
Range	5–16695	7–3088

**Table 5:** Summary of characteristics across healthy and benign cohorts. This includes sample number, age, BMI, ethnicity, benign conditions and CA-125 level. Adenomas include seromucinous adenomas, serous cystadenomas and mucinous cystadenomas. Myomas include lipoleiomyomas and leiomyomas. Fibromas include serous adenofibromas.

	Healthy Cohort (*n* = 35)	Benign Conditions Cohort (*n* = 12)
Sample types	Serum	Serum
Age—years		
Median	50	50
Range	36–61	36–71
BMI—no.		
Median	26	-
Range	22–30	-
Ethnicity—no. (%)		
Asian	0 (0.0)	12 (100.0)
Caucasian	35 (100.0)	0 (0.0)
Benign conditions—no. (%)		
Endometriosis	-	2 (16.7)
Adenomas	-	4 (33.3)
Myomas	-	2 (16.7)
Fibromas		1 (8.3)
Mucinous borderline tumor	-	3 (25.0)
CA-125—U/mL		
Median	-	25
Range	-	4–65

## Data Availability

Publicly available datasets were analyzed in this study. These data can be found here: http://www.cbioportal.org/study/summary?id=ov_tcga (accessed on 3 August 2021). The remaining data presented in this study are available within the article or on request.

## Supplementary Materials

The following are available online at https://www.mdpi.com/article/10.3390/ijms222011220/s1.
