# Supplementary material for: Identification of Tumor Antigens in Ovarian Cancers Using Local and Circulating Tumor-Specific Antibodies
Source: Int J Mol Sci. 2021 Oct 18;22(20):11220. doi: 10.3390/ijms222011220 (PMC8538754; doi:10.3390/ijms222011220)
Supplement: Supplementary file 1 [file ijms-22-11220-s001.zip › ijms-1382438-supplementary.pdf]

SUPPLEMENTARY TABLE

Table S1. Custom protein microarray antigen content. Antigens identified using the Benign-specific antigens are shown in italics.

**Antigen Name**	**CDI System ID**	**Antigen Name**	**CDI System ID**
ACVR2B	JHU15767.B18C29R12	MYLK	JHU14956.B13C15R58
ARPP21	JHU04803.B21C10R52	OR8D1	JHU06306.B7C8R40
ATP4B	JHU03952.B7C9R4	PAGE5	JHU10427.B11C2R44
AURKA	JHU17260	*PALM2*	JHU15444.B18C9R2
BAGE4	JHU12454	PDE4DIP	JHU06975.B8C3R54
CAMKV	JHU06536.B6C15R48	PEX19	JHU02653.B4C30R42
CCDC186	JHU08169.B12C8R12	PLEKHA8	JHU02463.B4C22R40
CROCCP2	JHU06009.B8C6R32	*PMEPA1*	JHU00953.B1C9R14
CTAG1A	JHU17795.B23C15R32	PNMA2	JHU15552.B18C6R2
CTAG2	JHU17338	PQBP1	JHU11866.B15C18R2
DDX53	JHU16271.B19C24R14	PRM2	JHU07753
DDX6	JHU16347.B18C18R14	PTMS	JHU11105.B12C13R54
DPPA3	JHU17798	RBM47	JHU16988.B18C13R60
HOXA1	JHU08864.B23C13R42	RNF31_frag	JHU17239.B22C16R46
IRF2BP2	JHU17814.B21C2R36	SERPINB1	JHU06687.B7C8R48
M0R1X1	JHU06623.B7C3R46	SHARPIN	JHU08807.B10C31R18
MAGEA10	JHU02350.B2C2R40	*SMYD5_frag*	JHU11589.B14C15R2
MAGEA12	JHU07448.B5C22R56	SPANXB1	JHU03149
MAGEA4	JHU01014.B1C24R18	STAT3	JHU03250.B2C22R54
MAGEA6	JHU13255.B15C16R26	TP53	JHU04788.B7C9R16
MAGEA9	JHU08970.B11C22R22	TP53	JHU13973.B14C27R40
MAGEB1	JHU04943.B6C30R16	UBQLN2	JHU09588.B9C21R26
MAGEB10	JHU15810.B19C8R10	UBQLN4_frag	JHU02783.B4C23R48
MAGEB4	JHU05618.B7C4R28	*USP14*	JHU02302.B1C10R32
MRFAP1L1	JHU02929.B2C1R46	XAGE3	JHU09595
